# Development of Neural Circuitry for Precise Temporal Sequences through Spontaneous Activity, Axon Remodeling, and Synaptic Plasticity

**DOI:** 10.1371/journal.pone.0000723

**Published:** 2007-08-08

**Authors:** Joseph K. Jun, Dezhe Z. Jin

**Affiliations:** Department of Physics, The Pennsylvania State University, University Park, Pennsylvania, United States of America; University of Massachusetts Medical School, United States of America

## Abstract

Temporally precise sequences of neuronal spikes that span hundreds of milliseconds are observed in many brain areas, including songbird premotor nucleus, cat visual cortex, and primary motor cortex. Synfire chains—networks in which groups of neurons are connected via excitatory synapses into a unidirectional chain—are thought to underlie the generation of such sequences. It is unknown, however, how synfire chains can form in local neural circuits, especially for long chains. Here, we show through computer simulation that long synfire chains can develop through spike-time dependent synaptic plasticity and axon remodeling—the pruning of prolific weak connections that follows the emergence of a finite number of strong connections. The formation process begins with a random network. A subset of neurons, called training neurons, intermittently receive superthreshold external input. Gradually, a synfire chain emerges through a recruiting process, in which neurons within the network connect to the tail of the chain started by the training neurons. The model is robust to varying parameters, as well as natural events like neuronal turnover and massive lesions. Our model suggests that long synfire chain can form during the development through self-organization, and axon remodeling, ubiquitous in developing neural circuits, is essential in the process.

## Introduction

Precisely timed sequential firing of neurons has been observed *in vivo* in a number of brain areas. A striking example is found in premotor neurons of the songbird zebra finch, which sings stereotyped song consisting of several repetitions of a motif, typically of 500 ms to 1 s duration; the projection neurons in the premotor nucleus HVC (used as a proper name)—believed to underlie the timing of song—spike sequentially at precise times relative to the motif during singing [1]. Visual cortical neurons in anesthetized cats [2] and cortical motor neurons in behaving monkeys [3] also exhibit spike sequences with precise timings spanning hundreds of milliseconds. Such sequences may serve as an infrastructure for learning temporally demanding tasks, such as well-timed motor actions and perceptual discriminations of temporal signals.

Theoretical studies [4]–[9] and experiments in cortical slices [2] suggest that sequential firings of neurons can be produced in networks within local brain areas. The topology of the synaptic connections between excitatory neurons, through which the spikes propagate, is critical to the production of sequences. The synfire chain, theorized first by Abeles [5], [8], is the canonical topology for sequence generation. Previous theoretical and experimental studies have shown that synfire chains are robust for spike sequence generation [10], [11]. They have also been proposed as the neural mechanism that underlies the precise spike sequences observed in the zebra finch premotor neurons [12].

**Figure 1:**
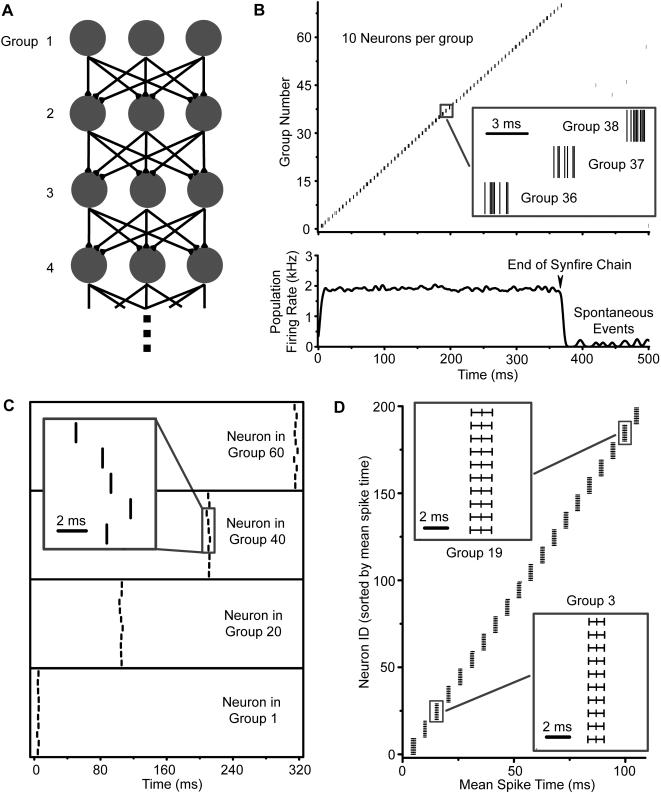
Synfire chain and its spike activity. (A) Topology of synfire chain. In a synfire chain, neurons (gray ovals) are organized into successive groups (shown as rows). Each group makes convergent synaptic connections (black arrows) onto the next. Group numbers are shown beside each group. (B) Single trial spike raster of population labeled by group number (upper), and associated population firing rate (lower). Here a group consists of 10 neurons. Each neuron spikes only once, and neurons in a group spike in tight synchrony. The inset shows a detail of the spikes from 3 successive groups. The population firing rate holds steady until the end of the chain, where it drops off to spontaneous levels. (C) Raster plots for select individual neurons across 10 trials. The lowest panel shows a neuron in Group 1, the starting group of the network; it is induced to spike by external input. Successive panels show neurons in higher groups, which spike due to the intrinsic synfire connectivity. The vertical alignment of spikes across trials suggests a high degree of temporal accuracy. The inset shows the details of the spike activity of a neuron in Group 40, which spikes approximately 200 ms after Group 1. (D) A raster plot showing mean spike times (vertical dashs) and spike time jitters (horizontal error bars) for the first 200 neurons across 1000 trials. Insets show the details of groups 3 (lower) and 19 (upper).

In the synfire chain architecture, neurons are organized into synchronous groups that make convergent feedforward synaptic projections onto successive groups (Figure 1A). With this topology, a neuron spikes only when induced to do so by the group of neurons that directly precede it. In this way, relative spike timing is preserved between groups of neurons. This is shown through an example simulation of a synfire chain in Figure 1B. The chain has 32 groups, each with 10 neurons. The spike raster shows the spike times of neurons for a single trial; the spikes from the same group are plotted on the same row. Individual neurons spike at very precise times relative to the activation of the chain across multiple trials (Figure 1C,D). In each panel of Figure 1C, we plot the raster for a single neuron across multiple trials; the group to which each neuron belongs is indicated in each panel. The vertical alignment of each spike across trials indicates the high reproducibility of individual neuron spike times in a synfire chain. Figure 1D is a summary of all neurons. Here, the mean spike time across 100 trials is shown as a vertical dash, and the standard deviation of the mean (jitter) as a horizontal error bar; note that it is possible to have millisecond accuracy.

The validity of synfire chains is still an active topic of debate, and an important unresolved issue surrounds their development. In particular, how is it possible to refine local neural circuitry so as to attain the high degree of synaptic specificity that is found in a synfire chain (Figure 1A). An attractive idea is that neurons self-organize into synfire chains through activity dependent plasticity of synapses. Activity driven refinement of *local* neural networks, through synaptic plasticity and axon remodeling, is ubiquitous in developing neural systems, and is a necessary supplement to the genetically programmed mechanism of laying out coarse connections between brain areas [13], [14]. Self-organization makes it possible to form refined connectivity with minimal guidance from external inputs. This is in contrast to a supervised learning mechanism for synfire chains, in which an external source repeats the same sequence of activity in order to entrain neurons to spike in a particular order [4], [15]. Supervised learning is an unlikely mechanism for developing neural systems, especially in motor areas, since both the fine-grained targeting of the external sources and their sequential activations are most likely absent. Songbirds do typically learn their song from an adult tutor [16], but here we are interested in the development of the fine-grained timing mechanism that underlies song. Our assumption is that this develops before song acquisition, which begins 30 to 40 days posthatch [16].

Previous theoretical studies of self-organized mechanisms for synfire chain formation have yielded mixed results. Hertz and Prügel-Bennett [17] implemented a firing rate-based Hebbian plasticity of synapses. They found that the mechanism leads to short chains with only a few synchronous groups. Recent works implementing spike-time dependent Hebbian plasticity rules [18], [19] yielded similar results [20], [21]. A more recent work [22] used no Hebbian plasticity, but instead applied both pre- and postsynaptic scaling for all neurons. In this model, a synaptic weight is updated to target the postsynaptic neuron activity to one spike per trial, and the amount of the update is proportional to the average activity of the presynaptic neuron. Using this strategy, a temporal sequence did emerge, but the network did not organize into a synfire chain. Consequently, the spike timings are not as precise as can be achieved by a synfire chain.

In this paper, we re-examine a self-organizing mechanism of synfire chain formation. We observe that the previous attempts omitted an important factor in developing neural circuitry—the activity-dependent remodeling of axon arbors. During development, the axon branches of a neuron undergo exuberant exploration in which many weak connections form to different postsynaptic targets; subsequently, they undergo remodeling in which most connections vanish and a few stable connections remain [14]. Examples where this process plays a critical role include the development of the neuromuscular junction [23] and the formation of ocular dominance stripes in cats [13]. Two-photon imaging studies show that axon arbor pruning and stabilization is intimately coupled with the maturation of synapses [24], [25]. The structural plasticity of axon arbors introduces constraints to the refinement process of neural circuitry, since the existence of a connection is a precondition to the change in synaptic strength. We explore the possibility that structural plasticity may be crucial for the development of temporal sequences in neural networks.

Our approach is to develop a model that allows neurons to self-organize into a synfire chain architecture using a Hebbian plasticity protocol—spike-time dependent plasticity—and axon remodeling, in which the formation of a finite number of strong connections from a neuron triggers pruning of the weak connections from it. For the former, we use an established phenomenological model [26], [27]. For the latter, we enforce a simple rule on all neurons—a limit on the number of “strong” synapses—to schematize the complicated biophysical process of axonal arbor maturation. Using a combination of the two strategies, we find it possible to form a synfire chain network from a randomly connected network. Our model assumes that neurons in the circuit spike spontaneously at low rates and that a subset of neurons, called the training set, is activated intermittently by an external source. In addition, we assume that neurons can silence or activate synaptic connections based on spiking activity. This last ingredient is important for maintaining excitatory balance in the network while allowing neurons to form connections to appropriate targets.

**Figure 2:**
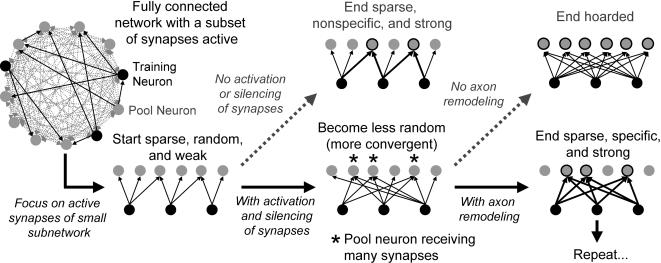
Cartoon of the formation model. The network is fully connected, but 90% of connections are silent synapses (gray dashed arrows). The active connections (black solid arrows) are randomly set. Black ovals are training neurons (TN), which receive external excitation at the start of each trial, and gray ovals are pool neurons (PN), which spike spontaneously. Following the bent black arrow shows a small subnetwork that includes only the TN and their active synapses and postsynaptic PN. Since the active network is sparse and random, TN do not converge upon the same set of PN except for a random few. Without the ability to turn on silent synapses (follow the gray dashed arrow to the upper middle subnetwork), STDP can act only over the active synapses. Therefore, only the few neurons receiving convergent synaptic input from the TN can spike consistently after the TN. If, however, silent synapses can activate due to spike activity (follow black solid arrow to the lower middle subnetwork), then the TN can activate synapses onto the same set of PN. Since these neurons receive more excitation and hence are more likely to spike, the synapses from the TN to these neurons are more likely to potentiate. This is a positive feedback. These synapses will pass the supersynaptic threshold (follow black solid arrow to the lower right subnetwork), and the TN will coordinate to make convergent synaptic connections onto the same set of PN. The TN do not connect to other neurons due to axon remodeling, in which weak connections from a neuron are pruned once a finite number of super-connections from the same neuron are formed. Without axon remodeling (follow gray dashed arrow to the upper right subnetwork), the TN can continue to activate synapses onto *all* PN and hoard the entire network to themselves, meaning that all neurons in the network will be induced to spike after they do.

The formation is characterized by a recruiting process in which neurons are added to a growing chain started by the training set, as depicted in Figure 2. Our model produces long synfire chains capable of generating spike sequences with timing accuracy on the order of milliseconds. We also find that the model is stable to neuron loss, either through one-by-one death during the formation process or through a mass “surgical”-like lesion of a mature network.

## Results

We aim to show that long synfire chains emerge in a network of spontaneously spiking neurons, through connectivity modifications driven by Hebbian synaptic plasticity and axon remodeling when a subset of them are activated intermittently by external inputs. We first give an overview of our model. Then, we describe the roles of each plasticity rule in the formation process. Finally, we present results from our model.

### Overview of Model

A cartoon of our model is shown in Figure 2; it depicts how different elements work together and also shows alternate scenarios if a particular element were removed from the model. For simplicity, we discuss the growth process as it occurs at the beginning of the synfire chain formation.

Initially, a neuron connects to all other neurons, but 90% of the synapses on these connections are non-functional (or “silent”, gray dashed arrows in Figure 2). Functional connections (thin black arrows) are sparse and weak, and their targets are randomly selected.

All neurons spike spontaneously at roughly 0.1 Hz due to noisy fluctuations of membrane potentials. Spike activity modifies all synaptic strengths, whether silent or active, through spike-time dependent plasticity (STDP) [18], [19]. A synapse remains silent if its strength does not exceed a threshold Θ*_A_*. With enough potentiation, however, a silent synapse can become active when its strength exceeds Θ*_A_*, and the opposite can happen if a synapse experiences too much depression. Because of spontaneous activity and STDP, synaptic strengths fluctuate, randomly activating and deactivating synapses; therefore, the functional connectivity of the network fluctuates, allowing patterns of connections that would otherwise be inaccessible in a static architecture. Synapses are also subject to an activity independent decay with a small rate. This discourages formation of reliable connections due to the spontaneous activity alone.

A subset of neurons is intermittently induced to spike synchronously by a brief external input. These neurons are referred to as “training neurons” (TN) (shown as black ovals in Figure 2), and the rest as “pool neurons” (PN) (gray ovals). The time between two activations of the TN defines a trial period (2 seconds of simulated time). Following the bent arrow from the full network leads to a subnetwork that includes only the TN and their active postsynaptic targets (Figure 2). Because connections are random between all neurons, they are nonspecific, meaning that there is no coordination between neurons to select the same set of postsynaptic targets.

A trial proceeds as follows. A brief burst of excitation, modeled as high frequency Poisson spike trains, induces the TN to spike. After the activation of the TN, some PN will be spontaneously active, and STDP will strengthen synapses from the TN to those PN. PN that have convergent connections from a subset of the TN are more likely to spike spontaneously after TN due to the depolarization of their membrane potentials, and hence are more likely to have the synapses from all TN strengthened; this is a positive feedback that makes the TN form strong connections with the same postsynaptic targets. The lower middle subnetwork in Figure 2 illustrates how the synapses are more specific due to the activation of synapses from the TN onto the same set of PN. This process, which we call “recruitment”, leads to a set of PN that have many afferent active synapses from the TN and can be reliably driven to spike by the TN.

It is expedient at this point to stress the importance of allowing activation or silencing of synapses. The upper middle subnetwork illustrates the consequence of removing this dynamic aspect from the network: STDP can strengthen only the existing static connectivity and therefore the TN will never connect to the same set of postsynaptic targets. Only those neurons that receive convergent connections at the outset (gray ovals with black outline in the upper middle subnetwork of Figure 2) will be recruited to spike after the TN. The lack of convergence from TN to PN is a consequence of the initial sparse connectivity. Though a more dense static connectivity removes the issue of convergence, it creates poor scalability and a high sensitivity to producing spike runaway in the network.

Another issue is the “hoarding problem” (shown in the upper right subnetwork of Figure 2). Since the TN always spike first in the network, they tend to strengthen, hence activate, synapses onto all neurons. Therefore, the TN will eventually hoard all of the PN if there is no restriction on the number of postsynaptic targets a neuron can have. Axon remodeling introduces such restriction. In our model, a neuron can emanate only *N_S_* connections with “supersynapses”; once this limit is reached, all other connections from the neuron are pruned. A synapse is super if its strength exceeds a second threshold Θ*_S_* (>Θ*_A_*). Axon remodeling, together with the tendency that TNs make converging connections to recruited neurons, limits the number of neurons recruited to the next group (the ones that spike after the TN) to approximately *N_S_*. The lower right subnetwork of Figure 2 shows the recruitment of the second group and the pruning of all other connections.

After the formation of the second group, the TN are “saturated”. The neurons in the newly formed second group replace TNs as the sites where PNs can be recruited. The external inputs can reliably activate the neurons in the second group. Thus, new PNs can be recruited to form a third group. The iteration of recruitment and axon remodeling leads to the emergence of a long synfire chain network.

Finally, there is global feedback inhibition in the network. It is important for both the replaying and the development of the synfire chain. Since all neurons can be spontaneously active at any time, they therefore can interrupt the playing of the chain. Global feedback inhibition discourages neurons from being spontaneously active while other neurons are spiking.

### Formation of synfire chain

**Figure 3:**
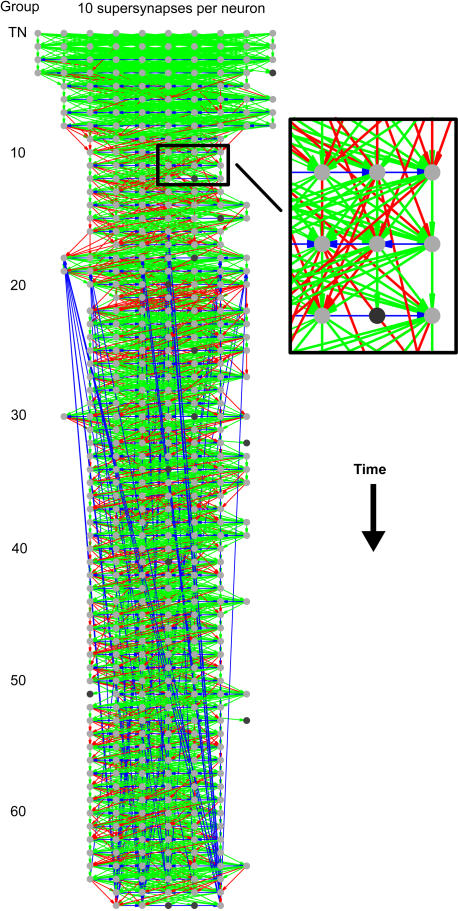
Topology of supersynapses of a developed network. Active synapses were originally laid down randomly with a connection probability 0.1. After 200000 trials, the neurons organize into a network that resembles a synfire chain (compare to Figure 1A). Only supersynaptic connections (arrows) and the neurons (circles) that receive them are shown. Light gray circles are saturated neurons; they have withdrawn their axons to all other neurons. Dark gray circles are unsaturated neurons; they have active subsuper synapses that are not shown. Green arrows are synapses that connect to neurons in the next group. Red arrows are synapses that connect to neurons in groups higher than the next. Blue arrows are synapses that connect to neurons in equal or lower groups. Neurons that are labeled in the same group are drawn horizontally in rows; these neurons fire near simultaneously. Successive groups are positioned vertically such that the relative spike time in the network flowing from top to bottom (see Text S1 for details of the algorithm used to assign groups). Each neuron had space to support 10 supersynapses. There were 10 neurons in the training set. Synaptic plasticity parameters for the simulation were set as follows. The LTP constant was *G_LTP_* = 0.3; the synaptic conductance threshold for activation/inactivation was Θ*_A_* = 0.2; the synaptic conductance threshold for supersynapses was Θ*_S_* = 0.4; the maximum synaptic conductance was *G*_max_ = 0.6 (the unit of all conductances is the leak conductance of a neuron). The rate of synaptic decay—the amount by which each synapse is scaled down after every trial—was *β* = 0.999996. See Materials and Methods for more details.

Using our connection plasticity model in conjunction with STDP, we find that it is possible to form a network with topology similar to a synfire chain, as shown in Figure 3. The figure shows only supersynaptic connections (arrows) and those neurons (ovals) that either receive or send one. Neurons are organized into groups starting with the training group (labeled T). Topology determines group membership of a neuron by counting the smallest number of synapses it takes to reach it starting from any of the training group; this group assignment is then corrected using a majority rule (see Text S1 for more details); therefore, the topology shown in Figure 3 should approximate the spiking order of neurons in the network. The color of arrows is a measure of its length in groups. Green arrows connect from the previous group to the next group; they are the kind of synapses one expects in a synfire chain. Red arrows go forward, but stretch multiple groups. Blue arrows connect neurons within group or even reverse in groups at any length. The grayscale of ovals determines whether a neuron is saturated or not. Light gray ovals are saturated—have only the supersynapses shown—dark gray ovals are unsaturated—have other subthreshold synapses not shown. Note that an ideal synfire chain would have only green synapses connecting light gray ovals with equal numbers of ovals per group; the network formed in our model is more general. In total, 443 neurons (out of the 1000 in the simulation) are organized into 67 groups. The network has 4410 supersynaptic connections; of these, 78 percent were forward connections (pointed to a higher group), 20 percent were lateral connections (pointed to the same group), and 2 percent were backward connections (pointed to a lower group)—note the long blue arrows in the network (these connections along with the lack of full recruitment of all available pool neurons will be discussed in the section titled Cycles). The network is shown at trial 200000. The saturation number of supersynapses was set to a constant 10 per neuron. See the figure legend and the Materials and Methods section for the value of other simulation parameters.

### Spike Activity of Developed Networks

**Figure 4:**
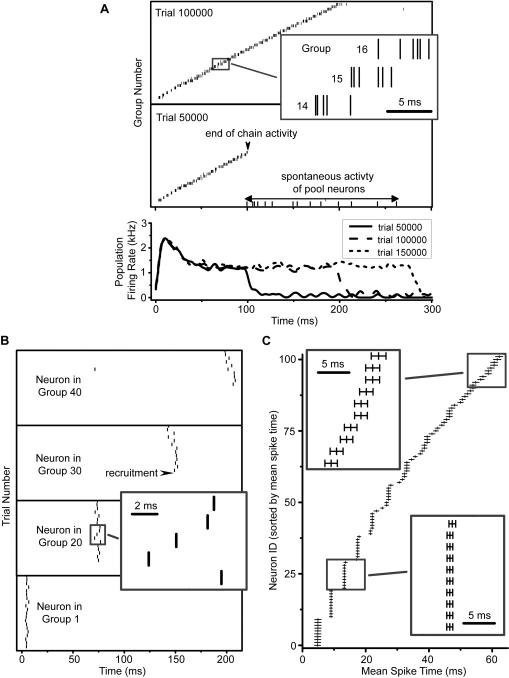
Spike timings of neurons in the network shown in Figure 3. (A) Raster plots (upper) show spike times of neurons for two different stages of the development. Spikes of neurons in the same group are shown in the same row. Spikes of all pool neurons are shown in the same row at the bottom. In trial 50000, there are 21 groups, and the chain activity lasts for approximately 100 ms, after which spontaneous activity of the pool neurons begins. By trial 100000, there are 39 groups, and chain activity lasts about 200 ms. The inset shows a detail of spikes from three successive groups; spikes of a group cluster together, but those of successive groups can overlap. The duration of the chain activity and its growth in time is also demonstrated with the population firing rate (lower). Spikes of all neurons were convolved using a Gaussian kernel with a standard deviation of 3 ms to compute the population firing rate. The firing rates for three different trials—50000, 100000, and 150000 (raster not shown)—are plotted versus time. The duration of chain activity increases linearly with the number of trials (data not shown). (B) Raster plots across trials for select neurons show how precise spike timings emerge. Each panel shows spike data across 300000 trials sampled every 10000^th^ trial for that neuron; the group to which each neuron belongs is indicated in the panel. The neuron in Group 1 is a TN, and therefore is induced to spike at the beginning of each trial (lower panel). The other neurons (upper panels) are recruited into the synfire network at later trials; thus, early on, they only spike spontaneously. As neurons from the chain strengthen synaptic connections onto each neuron, it begins to spike with high accuracy. Inset shows a detail from a neuron in Group 15. (C) Raster plot of mean spike times (vertical dashes) and spike timing jitters (horizontal error bars) for the earliest 100 neurons in the chain. The network—formed over 350000 trials—was simulated for an additional 1000 trials, and the spike data for all neurons was recorded. The first spike time of each neuron was averaged across all 1000 trials, and the jitter (standard error) of the first spike time was calculated. Only those neurons that spiked in at least one-half of all trials are shown. Insets show details from two different time periods. Note that neurons with smaller latency have smaller spike timing jitter.

The formation of a synfire-like topology does not guarantee that the network produces reliable spike sequences; the topology shown in Figure 3 is drawn to approximate the relative spike ordering in the network. Raster plots (Figure 4) confirm that the formed network is capable of precisely timed spike sequences. Figure 4A shows population raster plots during single trials labeled in the panels. For each panel, all spikes from neurons in the same group are placed on the same row. Note that successive groups fire in order. Comparing the developed network raster (Figure 4A) to the ideal synfire chain (Figure 1B), it is evident that groups of neurons in the developed network spike less tightly than those from the ideal network; this is unsurprising since connections that span across groups are possible in the developed network. The looser group activity also allows a more continuous spike time encoding as compared to the ideal chain, which spikes in discrete bursts. Figure 4A also shows how the network grows with developmental time. The number of groups grows linearly with the number of training trials until reaching a saturation in size (data not shown); the dynamics of the growth can be viewed in the movie (Movie S1) in Text S1; the saturation in size will be discussed in the section titled Cycles. Individual neurons spike with high accuracy across trials during training (Figure 4B); the plot shows raster data for five select neurons during the formation history (300000 trials sampled every 1000^th^ trial). Late recruited neurons spike with greater latency relative to the beginning of a trial, reflecting the group-by-group recruitment process. Most neurons in the chain can spike reliably and with a high degree of accuracy across multiple trials (Figure 4C); spike-timing jitters are on the order of a few ms.

### Cycles

**Figure 5:**
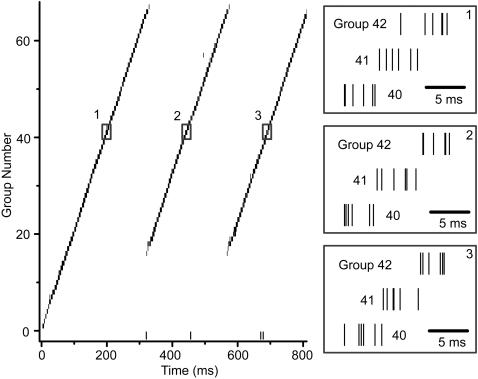
Cycles in spike activity. The raster shows activity in the network of Figure 5 later in development (Trial 300000). At this point, the network has developed a cycle that replays a portion of the chain activity. Insets show a detail of the same group of neurons in three consecutive cycles.

Around trial number 180000, the size of the developed network shown in Figure 3 plateaus at 67 groups. At that point, approximately half of the neurons have been recruited, while the other half remain in the pool. The growth ceases at this point because neurons at the end of the network form stable supersynaptic connections to neurons that are situated earlier in the chain (indicated by the long blue arrows in Figure 3); this represents a cycle. Cycles can occur because any neuron, not only pool neurons, may be spontaneously active—and hence recruited—after the developed network finishes spike activity. If one neuron is re-recruited to be a cycle, this may not be enough to reignite the chain activity by itself; nevertheless, the re-recruited neuron biases all of its postsynaptic partners—who are also in the chain—to be spontaneously active. Subsequently, those neurons are biased to be re-recruited to the end, forming a cycle; hence, in Figure 3 the long blue arrows converge upon the same targets. Figure 5 shows a raster plot of cyclic activity in the network at trial 300000. Note that in the 2^nd^ and 3^rd^ rendition of the activity, the spikes begin at the same group number.

**Figure 6:**
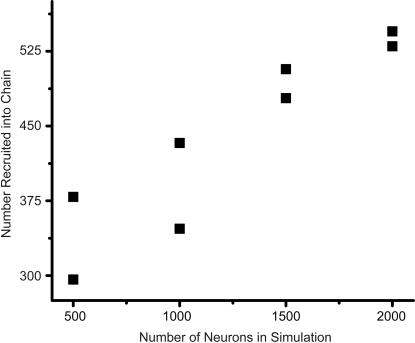
Size of developed network versus the total number of neurons. Simulations were repeated using 500, 1000, 1500, and 2000 neurons. The number of neurons that ended up in the chain were counted and plotted versus the total number of neurons. The relationship is positive.

Two factors limit the development of cycles: one, the spike refractory period which prohibits a neuron from spiking again for some time period, and two, long-term depression (LTD), which tends to weaken reverse synaptic connections. The neuron refractory period was set to 25 ms and the LTD time constant was set to 20 ms, making the LTD time window approximately 60 ms. Therefore, LTD defines a weak lower bound on the duration of synfire activity and hence size of the chain. In our simulations, we found typical spiking durations for a single cycle to be greater than 300 ms; therefore, the size of the developed network is not strongly constrained by these two factors. We ran an additional set of simulations to determine how the size of the network is related to the number of neurons in the pool (Figure 6). We found, as expected, a positive relationship between the two. Nevertheless, the relationship is weaker than linear, suggesting that other factors, like maximum synaptic strength, number of supersynapses, etc., may have a strong influence on the expected size of the network.

### Robustness to Parameters

**Figure 7:**
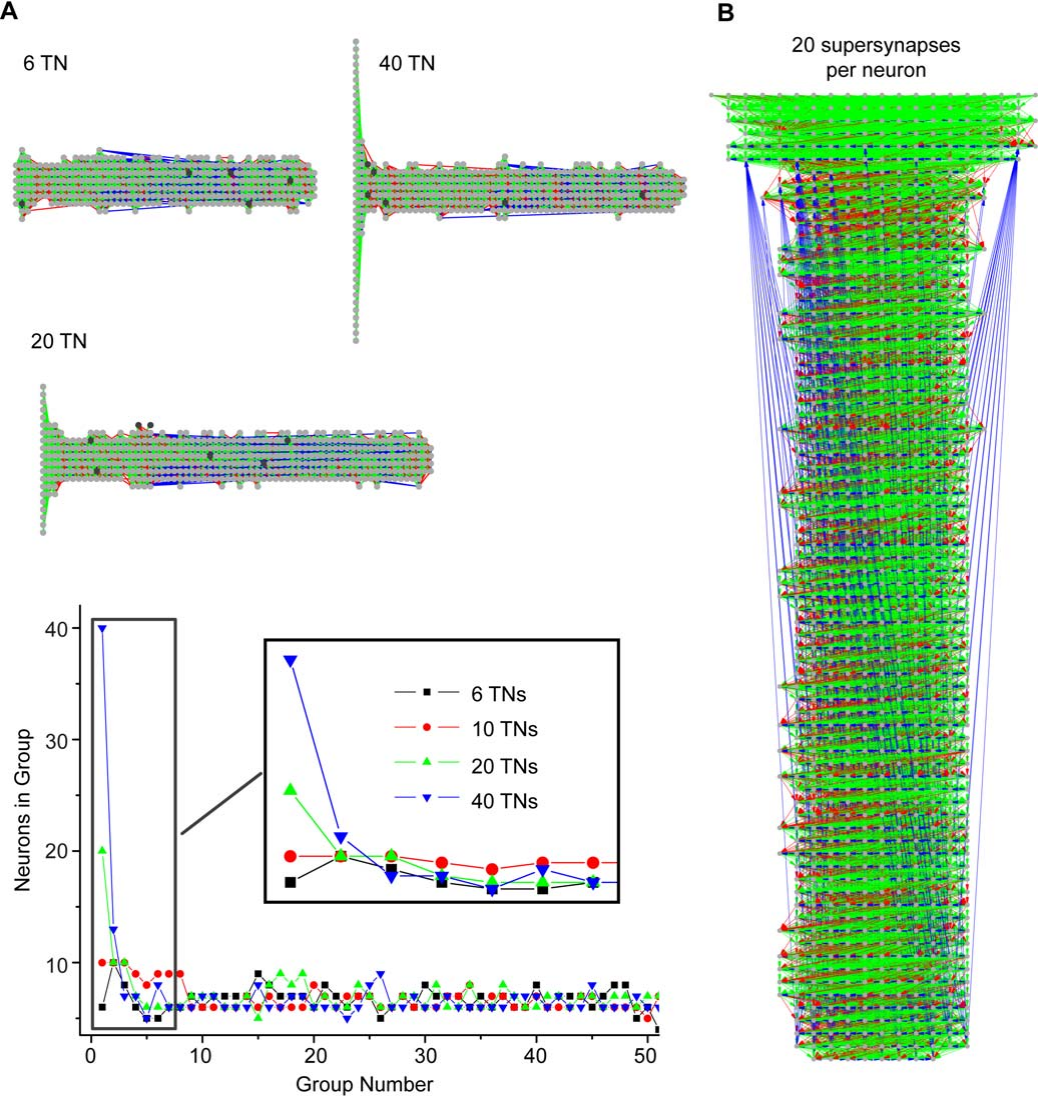
Simulations varying the number of training neurons and the number of supersynapses per neuron. (A) (Upper) Networks formed with three different numbers of training neurons (TN). After a few groups, the width of the synfire network returns to a steady state size. The color coding is identical to Figure 3. (Lower) The distribution of neurons in each group for four networks formed using different numbers of TN; line color and shape encode the different values of number of TN. The number of neurons per group quickly converges to the same number, independent of the number of TN. The inset shows the distribution for the first 7 groups. The curve with 10 TN is from Figure 3; the other three curves are from the networks above. (B) A network formed with the numbers of supersynapses and TN both set to 20; all other parameters were the same as in Figure 3. The major difference compared to the network shown in Figure 3 is that the number of neurons per group is higher.

**Figure 8:**
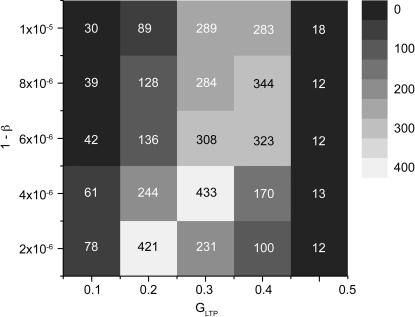
The network size as a function of the parameters *G_LTP_* and *β.* Each square represents a single simulation with the pair of simulation parameters indicated on the axes. For each point, the simulation was run until there were no further changes in the supersynaptic structure. Next, the simulation was run for an additional 100 trials, spike data was collected, and the number of neurons that spike in at least 75 percent of all trials was counted; this number, called the network size, is coded by grayscale and written in each box. A higher value indicates a longer synfire chain network since more neurons are induced to spike regularly.

Parameters used in Figure 3 do not have to be fine-tuned, as shown in Figure 7 and 8.

#### Training neurons

In Figure 3, we showed a network that formed using 10 TN, which also happened to match the number of supersynapses per neuron. Here we ran simulations where the number of TN was set to 6, 20, and 40. Figure 7A (upper) shows the supersynaptic topologies of the three developed networks; color coding is identical to that of Figure 3. Note that only the first few groups of each network show any significant difference in structure. Figure 7A (lower) demonstrates this by plotting the number of neurons per group as a function of group ID number. Regardless of the number of TN, the chain rapidly converges to a steady-state value; the line representing 10 TN is from Figure 3. We found that the minimum number of TN required for this particular set of parameters was 6, which is roughly the number of presynaptic neurons needed to fire within close temporal proximity in order to make a postsynaptic neuron spike.

#### Number of supersynapses

It is also possible to change the number of slots for supersynaptic connections on each neuron (Figure 7B). Leaving all other parameters the same, we increased the total number of supersynapses per neuron (and number of TNs) from 10 to 20. After 200000 trials, the network forms a synfire chain like that shown in Figure 3. The major difference is that the network shown here has more neurons per group.

#### Synaptic plasticity parameters

There are two main parameters to synaptic plasticity: the LTP constant, *G_LTP_*, which determines the rate of potentiation; and the homosynaptic depression rate, *β*, which determines how rapidly synaptic values decay each trial. Figure 8 shows how the network size varies as a function of these two parameters; all other parameters were held fixed (10 TN and 10 supersynapses). For each parameter pair (25 pairs are shown) a 1000 neuron network was simulated until the network topology ceased to change significantly. Most simulations terminated after 250000 trials; others lasted 500000 trials or more; the minimum number was set to be 100000 trials. After the formation completed, the simulation was run for another 100 trials. All neurons that spiked within the first 1000 ms in at least 75 percent of the trials were counted; this quantity indicates the number of neurons that are driven to spike reliably in the chain and is called the size. Figure 8 shows a band where the chain sizes are large, suggesting that the model can handle changes to *G_LTP_* and *β*. The shape of the phase diagram can be understood in the following way. If *G_LTP_* is high, synapses can potentiate beyond the superthreshold through spontaneous activity alone. This eliminates the need for cooperation amongst neurons in the same group; they will not converge upon the same postsynaptic targets, and the long chain does not form. This condition is shown in Figure 8 when *G_LTP_* = 0.5. At the other end, when *G_LTP_* is small, the synaptic strengths cannot maintain high values, and the chain is also short in length; this effect is shown in Figure 8 when *G_LTP_* = 0.1. If *β* is low hence synaptic decay rate is high, synapses tend to maintain values below the superthreshold and the chain does not form. This effect is shown when 1-*β* = 1×10^−5^ in Figure 8. On the other hand, when synaptic decay is low, supersynapses tend to form spontaneously. In Figure 8, one sees that the chain is longer for *G_LTP_* = 0.4 when *β* takes on an intermediary value (1-*β* = 8×10^−6^ and 1-*β* = 6×10^−6^ in Figure 8). Between the extremes mentioned above, the chain grows into long sequences.

### Turnover and Lesions

Real neural networks must be robust to the loss and renewal (turnover) and mass loss (lesions) of neurons. For example, projection neurons in the songbird premotor nucleus are known to turnover in developing and adult songbirds (see review in [28]). To test our model against these effects, we simulated both kinds of neuronal loss. First, we simulated neuronal turnover by assuming that neurons “die” and are “born” randomly through a Poisson process during network formation. Second, we simulated a surgical lesion of the brain area by “destroying” some percentage of the neurons in a mature network and observed its recovery. See the Materials and Methods section for details.

**Figure 9:**
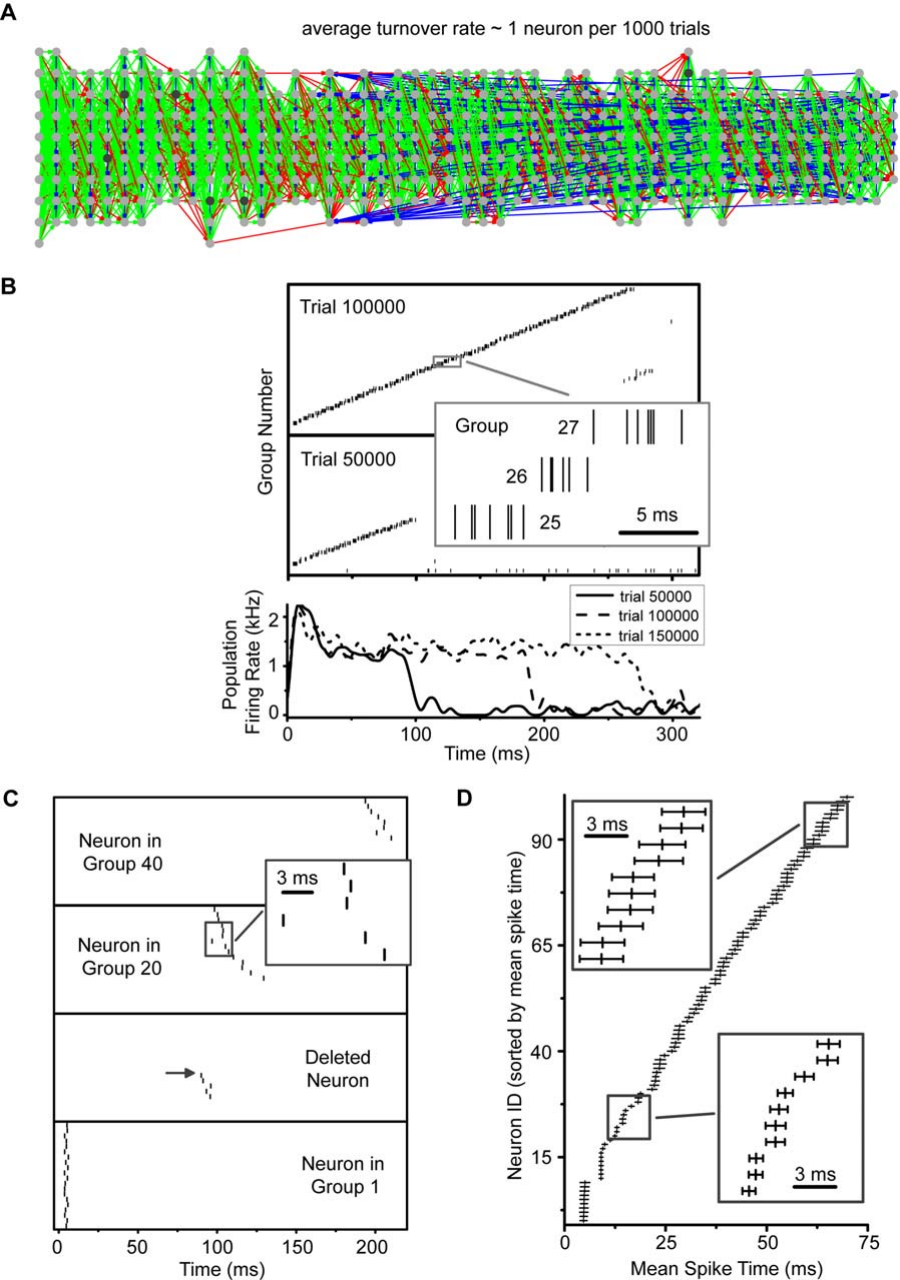
Network formation with turnover. (A) A network formed while neurons died and renewed at an average rate of 1 per 1000 trials through a Poisson process. Even with the turnover, the neurons were able to form a synfire network. (B) Population activity in single trials during the formation process are similar to those without turnover (Figure 4A). The duration of chain activity increases with the number of training trials. (C) Spikes of individual neurons across trials show different behaviors than those without turnover (Figure 4B). A recruited neuron can be deleted (second panel from bottom). Upstream neurons (three upper panels) can shift their spiking times forward as they fill slots vacated by deleted neurons in earlier groups. (D) Spike timings across 100 trials shows that neurons in the chain spike with accuracy on the order of ms.

In Figure 9A, we show a network that developed with an average neuron turnover rate of one death and renewal every 1000 trials; all other parameters are identical to those used in Figure 5. Even with neuronal death, the model can form a long stable chain. The network here is shown after 250000 training trials; during that time, 230 neurons were killed and renewed, a little less than one-quarter of the total in the simulation.

Spike activity in the network is also sparse and temporally precise. Chain activity increases with the number of training trials (Figure 9B); network activity shows little difference from when there is no turnover. The spike history of individual neurons tells a different story (Figure 9C). After recruitment into the synfire network, each neuron can spike at a precise time; the mean spike time, however, can drift forward as training proceeds. The reason is that as neurons downstream in the synfire chain are deleted (second panel up from bottom in Figure 9C), neurons upstream are more likely to fill vacancies downstream. These neurons have an advantage over free pool neurons because they are induced to spike, and therefore their afferent synapses from downstream neurons LTP at a higher rate than synapses onto pool neurons do. Despite the drift, over shorter numbers of trials, the mean spike time of each neuron has high accuracy (Figure 9D).

**Figure 10:**
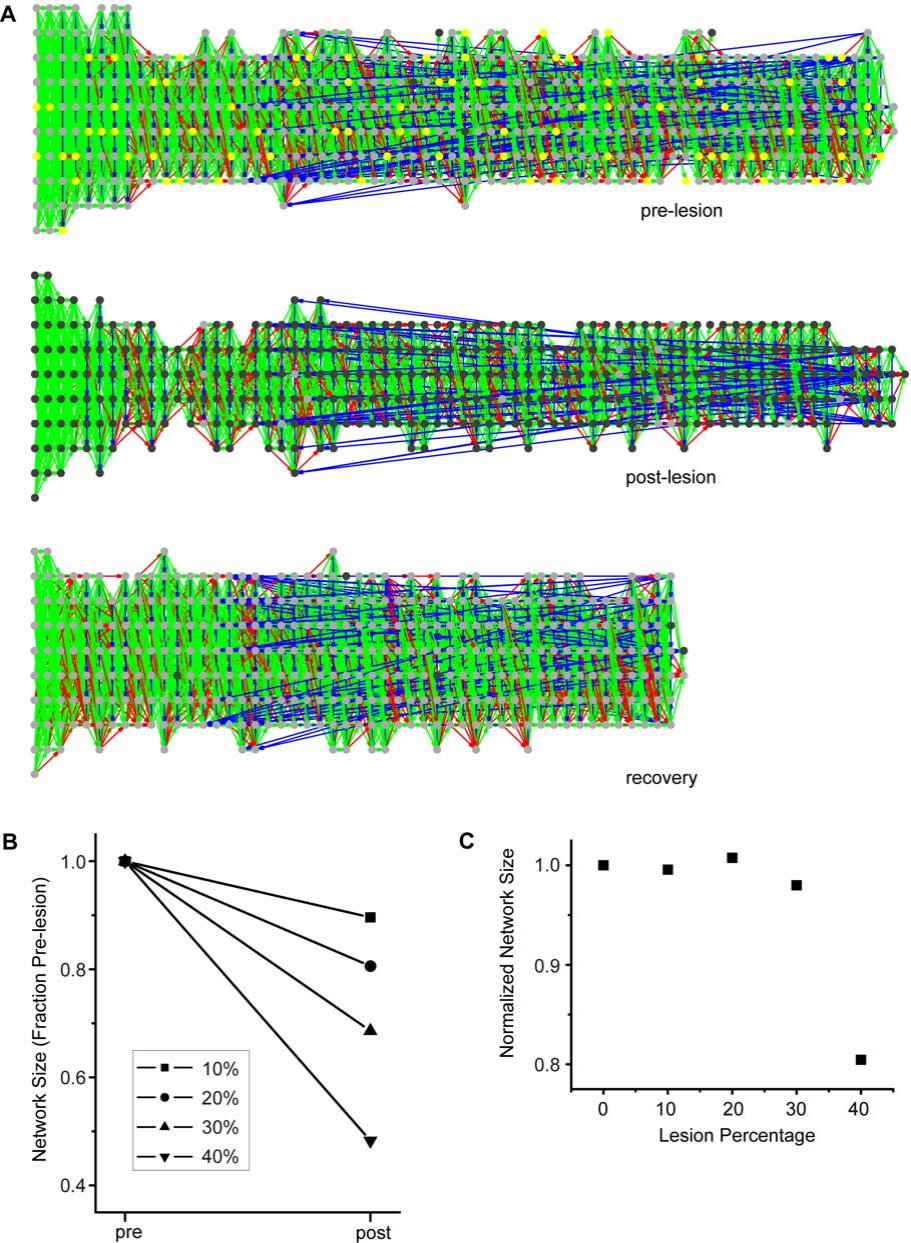
Network recovery from a mass lesion. (A) The mature network of Figure 4 (upper) was given a 20% lesion (middle). The formation process was then allowed to proceed as normal, with no further neuronal death. The network was able recover after 100000 trials (lower); it, however, ended up shorter and wider than normal. (B) Four different simulations using the same base network, but performing different levels of lesions at 10, 20, 30, and 40 percent. The plot shows the change in size of the chain, as defined by the number of neurons spiking reliably, from pre-lesion to post-lesion recovery. The chain does not recover to its normal size; it is shortened. (C) The size of the post-recovery network normalized by the number of neurons left intact directly after the lesion. The normalized size is close to 1 for lesions less than 40%, indicating that neurons are not added nor lost during the recovery. At forty percent however, the normalized size dips below 1, indicating that additional neurons are lost during the recovery period.

Besides one-by-one neuronal death, which occurs naturally in the brain, our mechanism is also robust to more massive deaths of neurons as might occur in head traumas or from surgery. We performed simulations, where we took the already developed chain from Figure 4, and then randomly killed a percentage of the neurons (Figure 10). The upper network of Figure 8A shows the pre-lesion network with those neurons chosen to die colored yellow. The middle network of Figure 10A shows the post-lesion network right after the (∼20%) lesion. The lower network of Figure 10A shows the network recovery after 100000 training trials. The final chain is both shorter (smaller in total number of groups) and wider (greater in number of neurons per group). Note that the cycle from the pre-lesion chain persists after recovery.

We repeated the simulation using different levels of lesions (Figure 10B). Different amounts of lesions lead to recovered networks of different sizes; in all cases, the chains were shortened (Figure 10B). The relative amount that the chains were shortened did not depend on the level of lesions to a point (Figure 10C). For 10, 20, and 30 percent lesions, the networks were shortened by 10, 20, and 30 percent respectively. For a 40 percent lesion, however, the network shortened by a disproportionately large amount. This means the chain maintains its relative size during recovery up to some point of neuronal loss; beyond that point, it loses additional neurons.

## Discussion

Neural circuits that generate precisely timed spike sequences can serve as an infrastructure for learning motor controls or sensory discriminations that require precise timings; neurons in such networks are time markers to which actions or sensory inputs can associate. Our model suggests a mechanism for the formation of synfire chains during circuit development. The process is driven by intermittent activations of a subset of neurons, which, along with the spontaneous activity, drive modifications of connections between neurons through synaptic plasticity and axon remodeling.

Axon remodeling is a key ingredient of our model. Initially, a neuron contacts many postsynaptic targets with weak or silent synapses. Such exuberant connections make it possible for synchronously firing neuron groups, like the training neurons, to find new recruit neurons to add at the end of the existing chain. The strengthening rate of synapses is not equal. Synapses on a pool neuron that receive convergent connections from a large fraction of a synchronously spiking group that is already in the chain tend to be strengthened more rapidly; for example, pool neurons selected for the second group were initially contacted by many training neurons. In our model, a neuron supports only a finite number of strong connections; once the number is reached, all other weaker connections are pruned. Such maturation-triggered pruning is crucial for preventing all neurons from being recruited into the second group; without it, all connections from the training neurons, however weak initially, are eventually strengthened to maturation due to the consistent activations of the training neurons and their strongly connected targets. Saturated neurons, with the allocated number of strongly connected targets, do not form further connections. Thus, only a finite number of neurons are recruited into the second group, with the number of neurons in each group roughly equal to the number of allowed strong connections from a neuron. After formation, the second group replaces the training group as the active zone, to which the pool neurons are connected to form the next group. This process iterates, and leads to the formation of long synfire chains. It is important to note that our model uses cues that are local to an individual synapse or to a single neuron; no global information about the network is necessary.

In our model, axon pruning is triggered by competition between the axon branches of the same neuron; once a finite number of branches form strong synaptic connections to their targets, all other branches are pruned. There are no experiments yet directly demonstrating this mechanism; however, evidence can be inferred from several recent experimental results. Recent two-photon imaging experiments that followed axon dynamics demonstrated that the stability of axon branches of a neuron is closely linked to the formation of strong synapses: branches with mature synapses are stable, whereas those with weak or no synapses are prone to retraction [24], [25]. Moreover, consistent activation of the neurons enhances the stability of branches that have strong synapses while concurrently inducing retractions of those that have weak synapses [25]. This supports the idea that maintaining a finite number of strong connections discourages formation of additional strong connections.

Activity dependent pruning of axons is usually linked to the competition between branches from different neurons to innervate a postsynaptic target. The axon branch from the most active neuron usually wins, which leads to retraction of axons from other neurons. This mechanism has been observed in retinal ganglion cells [29], and most extensively, in motor neurons [30]. One motor neuron can innervate many muscle fibers, but a muscle fiber can be connected by only one motor neuron. This connectivity is formed through competition between axons from different motor neurons at the neuromuscular junction, where axons that are most active usually win. However, axon retraction is not entirely determined by such inter-neuronal competition, as demonstrated by several experiments [31]–[33], which observed axon retraction even after the level of competition was reduced by removing a bulk of motor neurons and leaving abundant muscle fibers to innervate. That a certain amount of the axonal withdrawal may be intrinsic to the motor neuron is also shown by a recent experiment [23], which suggests that the competitive vigor of the axon branches of a neuron is reduced as some of its branches win. This prevents the undesirable and never observed outcome that a single motor neuron with the “best activity pattern” wins all the competitions and innervates an enormous number of muscle fibers [23]. These results, taken together, support the idea of competition between axon braches of the same neuron, probably due to a limited resource for maintaining strong synapses [23]. Such competition has been demonstrated in hippocampal neurons [34].

Inter-neuronal competition generally restricts the number of afferent synapses onto a single neuron, which is the case for the neuromuscular junction. This restriction alone does not help to avoid the formation of short chains in our case, since it does not limit how many strong connections the training neurons make. We did not explicitly limit the number of afferent synapses onto a single neuron; nevertheless, our model avoids an undesirable state, where a single neuron receives a large number of connections, in a natural manner; once a neuron is completely recruited into the chain, it is unlikely—although not strictly prohibited—that any more neurons will make a synapse onto the newly recruited neuron; this is because only the neurons spiking before the new recruit can LTP consistently onto it; earlier neurons, however, are saturated and cannot make new synaptic connections.

It is likely that both inter and intra neuronal competitions are important for axon remodeling. In our case, the inter-neuronal competition is not necessary but the intra-neuronal competition is. Regardless of the exact nature and implementation of remodeling, we have found that it could lead to the formation of sparse and precise temporal sequences. Indeed, we found that using Hebbian synaptic plasticity alone leads to instabilities in network activity; spike activity either decayed rapidly or exploded. Axon remodeling mitigated these instabilities. Axonal withdrawal ensured that neurons would not excite neurons unnecessary to sequence generation, thereby removing the instability towards over-excitation.

“Switching on” of silent synapses is important for the formation of convergent strong connections from neurons of the same group to a neuron in the next group. The reason is that, once a neuron is consistently activated by a subset of synchronously active group of neurons, all connections from the group will be strengthened to maturation, even the ones with initially silent synapses. We apply the term “silent synapse” loosely in this context. The key feature of our model “silent synapse” is that it represents a potential functional synaptic connection between two neurons. There are at least four experimental models explaining the switching on of putative silent synapses [35]. Our model does not depend critically upon which one (if only one) in the end is correct. The critical point for our purposes is that two neurons which have no functional synaptic connections can develop them through activity. In our case, for computational simplicity, we allowed silent synapses to undergo the same LTD and LTP induction as active synapses. It is unclear that this should be the case. In our model, however, the tracking of “synaptic strength” for silent synapses is merely a marker for the level of correlation between two neurons. The details of this marker may be quite different than what occurs in STDP, but our model again should not depend critically on those details. The one detail which may matter is whether the subthreshold rule is antisymmetric in time. Subthreshold LTD suppresses the activation of synapses that could make short cyclic (back) connections in the network. If the rule were symmetric in time, then postsynaptic neurons would tend to activate synapses back onto neurons that fire shortly before them. This effect is easily mitigated so long as neurons have a refractory or adaptation period that is on the order of the time constant that correlates two neurons' activities. Recently, Shen et al. [36] reported that activation of silent synapses is asymmetric; silent synapses in cultured hippocampal neurons activated when the stimulation was applied to the presynaptic neuron only and not when applied to the postsynaptic neuron only. Since silent synapses are thought to be mediated through NMDA receptors (see reviews in [37], [38]), the asymmetry of the time rule is not inconceivable.

Silent synapses also provide the possibility of connecting any pair of neurons while avoiding spontaneous runaway excitation in the network. An alternate proposal is that a large number of synapses begin with zero weight, but are active as soon as they LTP above zero. Though this situation can lead to the formation of short synfire chains, which we confirmed in simulation, it has undesirable side effects such as poor scalability and an instability towards synchronizing the entire population of neurons; this is due to the nature of the all-to-all connectivity which makes it difficult to control excitation—even if most weights are small, the cumulative effect can be large, especially when the number of neurons is large.

In our model, synaptic strengths decay with a small constant rate (homosynaptic depression). Homosynaptic depression prevents formation of strong connections between random pairs of neurons simply due to spontaneous activity. Without it, random potentiations can accumulate and eventually lead to strong synapses. Although homosynaptic depression has not been emphasized as an important form of synaptic plasticity, its existence can be inferred from the fact that AMPA receptors, which are major transmitters of excitatory synaptic currents, are constantly internalized and degrade [39]; consequently, the efficacy of a synapse is reduced constantly unless it is consistently potentiated.

The network formed by our model is similar to a synfire chain [5], [8]. Neurons newly recruited into the network are added to the end; therefore, the model predicts that learned sequences should grow gradually larger as the sequence forms. The model also predicts the appearance of cycles in the chains. This is not undesirable, since sequences such as birdsong are known to consist of a few introductory notes followed by a series of repeated motifs. The appearance of cycles suggests that the same set of neurons encodes repeated motifs. The growth of network is terminated by the formation of a cycle, and approximately one third to half of the neurons are incorporated into the network. Increasing the total number of neurons leads to longer chains. The rest of the neurons remain in the pool, and do not spike at precise times. This result is consistent with two observations on HVC of zebra finch, in which a synfire chain-like network is proposed to underlie the generation of the precise spike timings of the projection neurons [12]: about 60% of projection neurons are active during singing [40], [41]; and the durations of motifs sang by individual zebra finches are positively correlated with the sizes of HVC [42]. We tested our model to a variety of conditions. The formation of a synfire like network with sparse precise spike sequences was robust to those conditions. We also tested the model to natural events such as neuronal turnover and lesions. In both cases, we found that the model was still robust. Neuronal turnover did not leave any noticeable deleterious effect on the formed chain. It did, however, change the formation process, as neurons gradually shifted their mean spike time in response to the loss of neurons. Therefore, neurons downstream of the sequence circuit could see a gradual change in the sequence. In HVC, projection neurons are constantly renewed [43], [44]. The renewal-induced changes in the sequence could be useful for novelty and invention as is seen in birdsong, especially during the song learning period when the turnover rate is high. In the case of lesions, we found that the damage by the lesion could be “frozen” into the network, even though the total number of neurons is kept the same in the recovery phase as before the lesion; the recovered network might be operable, but nevertheless impaired. The main effect is that the length of the chain is reduced after the recovery, and the amount is proportional to the percentage of neurons lost. Scharff et al [45] induced death of HVC projection neurons in zebra finch, and observed variable degrees of recovery of the songs even though the incorporation rate of new neurons is increased. This work did not correlate the amount of lesion to the degrees of song recovery, and did not report on the effect on the motif length. More quantitative experiments are needed to address this issue. In our model, small amount of lesion (<10%) does not severely affect the network function even at the onset of the lesion. This is due to the redundancy of the connectivity between the groups. After recovery, the length of chain is reduced accordingly (<10%). A recent lesion study that induced less than 10% lesions in zebra finch HVC observed that songs recovered within 2 weeks [46]. The motif length did not change significantly. The process of song recovery is likely due to many factors beyond the timing network in HVC [46]. Our model does not describe how syllable number and structures recover. However, it does predict that such lesion should lead to shortening of the motif on a longer time scale of recovery, perhaps on the order of a few months. Increasing the level of lesion to 20% might be required to clearly see the effect.

In conclusion, we have shown that long synfire chains can form through a self-organization process. The connections between neurons are modified through STDP of synapses, axon remodeling, and synaptic decay. Driven by intermittent activations of a subset of neurons and spontaneous activity, a long chain network emerges through a group-by-group recruitment. Our results demonstrate that synfire chains can emerge during the development, and can serve as an infrastructure for learning timing-dependent motor or sensory functions.

## Materials and Methods

### Network

We simulate a network of 1000 recurrently connected excitatory spiking neurons with global feedback inhibition. The inhibition is mediated directly; a single inhibitory spike is delivered to all excitatory neurons whenever any excitatory neuron spikes. Dynamics of the network are run in trials with a fixed duration of 2000 ms in simulated time. At the beginning of each trial, neuron variables are randomized and the spike histories are reset—trials are *not* contiguous segments.

### Leaky Integrate and Fire Neurons

We use leaky integrate-and-fire unit to model each spiking neuron. Subthreshold membrane voltage evolves according to
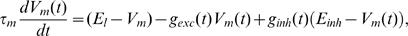
Where *τ_m_* = 20ms is the membrane time constant; *V_m_* is the membrane potential in mV; *E_l_* = −85 mV is the leak reversal potential; *g_exc_ (t)* is the excitatory conductance due to all excitatory synapses; *g_inh_ (t)* is the inhibitory conductance from all inhibitory synapses; *E_inh_* = −75 mV is the reversal potential of the inhibitory synapses. Here the leak conductance has been set to unity, and all synaptic conductances are measured relative to the leak conductance; this normalization is implied throughout. If the neuron membrane potential depolarizes to *V_thresh_* = −50 mV, the neuron emits a spike with 2 ms latency. After a spike, the neuron resets its membrane potential to *V_reset_* = −80 mV and enters a hard refractory period of 25 ms. The long refractory period enhances stability of spike propagations in the synfire chain network [12]. The parameters are chosen to roughly match the properties of a two-compartment model of premotor projection neurons in songbirds, which is conductance based and includes both somatic and dendritic compartments [12]. The precise values of the parameters are not important. We have confirmed that synfire chains also form with the two-compartment model (data not shown).

### Synapses

Each neuron tracks two total synaptic conductances, one inhibitory, *g_i_*, and one excitatory, *g_e_*. Synapses follow “kick-and-decay” dynamics. When a postsynaptic neuron receives an excitatory (inhibitory) spike, the excitatory (inhibitory) conductance discontinuously jumps *g*(*t_n_* ^−^)→*g*(*t_n_* ^−^)+*G*, where *g*(*t_n_* ^−^) is the synaptic conductance just before the arrival of the spike and *G* is the synaptic strength of the incoming spike. In between spike arrivals, the synaptic conductance decays exponentially with time constant 5 ms for excitatory synapses and with time constant 3 ms for inhibitory synapses.

### Excitation

There are two sources of excitation in the network, neuron-to-neuron interactions and background spontaneous activity. The former are represented in the synaptic weight matrix *G_mn_*, which gives the synaptic strength at which a presynaptic neuron *m* connects to one of its postsynaptic neurons *n*. The latter are delivered through a Poisson spike train with a frequency of 40 Hz and amplitudes for each spike uniformly distributed from 0 to 1.3.

### Inhibition

There are two sources of inhibition in the network, one from a global interneuron and another from background spontaneous activity. The global interneuron acts in the following simplified manner: for every excitatory spike from any neuron in the network, it emits a single inhibitory spike back to all neurons using a constant inhibitory conductance *G_inh_* = 0.3. We chose this method to reduce computational load. Though it is not a realistic implementation, for the purposes of our model, it is sufficient. The primary role of inhibition in our network is to discourage spontaneous activity during the running of the existing chain. Aside from the feedback inhibition provided by the global interneuron, there are also spontaneous inhibitory spikes that are delivered through a Poisson train of frequency 200 Hz and amplitudes uniformly distributed between 0 and 0.1.

### Spontaneous Activity

The combination of background excitation and inhibition generates membrane fluctuations with a standard deviation of approximately 7 mV, and it is enough to drive each neuron to spike at frequency ∼0.1 Hz.

### Spike Timing Dependent Plasticity (STDP)

For the Hebbian plasticity mechanism, we use STDP [18], [19] on excitatory synapses. The method applied here is adapted from the models found in [26], [27]. Synaptic strengths update according to the precise spike timing of the pre- and postsynaptic neurons; the STDP kernel used in the simulation is shown in Text S1 (Figure S1). We implement the STDP protocol in the following manner. Whenever a neuron spikes, all afferent synapses onto the neuron undergo LTP and all efferent synapses undergo LTD.

More specifically, consider a neuron pair *k* and *m*, with neuron *k* a presynaptic neuron to neuron *m*. Now, say neuron *m* spikes at time *t_m_*, then the synapse *G_km_* undergoes LTP at time *t_m_* by an amount that depends on the spike time history of neuron *k*, given by
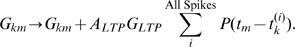
Where *G_LTP_* is the LTP strength, and *A_LTP_* = 0.01 determines the maximum fraction of *G_LTP_* that a synapse can increase by per spike pair. The actual amount of LTP for a given spike pair is given by their exact spike times through the potentiation curve *P(Δt)*, where Δ*t*≥0. The potentiation curve is given by
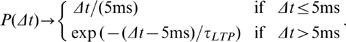
In other words, the potentiation curve rises linearly when spikes are within 5 ms and decays exponentially beyond that time with the LTP decay time constant *τ_LTP_* = 20 ms, which determines how rapidly spikes in the past are “forgotten” by LTP. All synaptic strengths are capped by the same maximum value *G*_max_. If LTP causes the synaptic strength to go above the maximum, the strength was set to *G*_max_.

Now consider a second neuron *n* that is a postsynaptic neuron on neuron *m*, then the synapse *G_mn_* undergoes LTD at time *t_m_* by an amount that depends on the spike time history of neuron *n*, given by
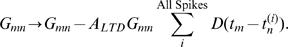
Where *A_LTD_* = 0.0105 is the maximum percentage of *G_mn_* that the synapse can decrease per spike pair—note that LTP is based on a constant value whereas LTD is based on the current synaptic strength; the depression curve *D(Δt)*, Δ*t*≥0, determines the amount of LTD. It is given by
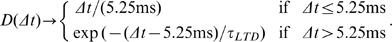
The depression curve rises linearly when spikes are within 5.25 ms and decays exponentially beyond that time with LTD decay time constant *τ_LTD_* = 20 ms. The value of *A_LTD_/A_LTP_* = 1.05 was set to match the value from Song et al [26].

### Homosynaptic Depression

All synaptic strengths decay at a slow constant rate. At the end of a trial, each synaptic strength is replaced using the rule *G_nm_*→*β*·*G_nm_*, where *β*<1 but very close to 1. The homosynaptic depression rule is a slow memory leak in the system.

### Training

Spontaneous activity alone does not lead to a synfire chain using the above rules of synaptic plasticity. The reason is that neurons are not associated consistently in groups or in sequences. Also, the activity has no consistent start point; therefore, if any sequences do develop, they can only be accessed by waiting for the correct random stimulation. At the minimum, a training protocol should define the start of the sequence. This is done by selecting a subset of neurons, called training neurons (TN), that receive strong excitatory external input, inducing them to spike synchronously at selected times. In our case, the external input arrives at the beginning of each trial in the form of high frequency (1.5 kHz) strong amplitude (2.0) Poisson spike trains that are 8 ms in duration. This is sufficient to drive each TN to spike once with a jitter of approximately 1 ms. This is the only activity imposed on the network; all neurons, including TN, spike spontaneously throughout the trial.

### Silent Synapses

To model the exuberant connection phase, we allow neurons to make all-to-all synaptic contacts. Only a small percentage of these, however, are active (initial probability = 0.10). The others are functionally silent, meaning they do not produce a physiological effect on the postsynaptic target. A synaptic “strength” is tracked for all synapses, and STDP is applied to both kinds. Synapses transition between either state by crossing a threshold, Θ*_A_*, called the activation threshold; silent synapses become active by going above threshold; active synapses become silent by going below threshold. With the modulation of silent synapses into the active state, it is possible for TN with divergent connections to converge upon the same postsynaptic targets. It is also possible for the postsynaptic target to silence synapses onto its presynaptic neurons; in this way short cycles are discouraged in the network. Figure S2 in Text S1 shows how silent synapses work in the simulation.

### Axon Remodeling

Neurons in our model withdraw axons when they have enough synapses that are of sufficient strength. We do this by means of a second threshold, Θ*_S_*, called the super threshold; any synapse going above this threshold is labeled a supersynapse. Each neuron is given a limited number of slots for supersynapses. When that number is reached, the neuron saturates and withdraws all other synapses. Withdrawn synapses do not produce physiological effects onto their targets, nor do they continue to undergo STDP. They do, however, continue to decay in “strength” through the homosynaptic depression. Since the axon withdrawal is reversible—if one of the supersynapses dips below Θ*_S_*—continuing to track the strength is a way for the system to have some memory of its past configuration, though that memory should fade in time if the neuron remains saturated for a long time. This method was chosen because withdrawals in the network occur instantaneously rather than through a more realistic gradual process. Reversal is rare during development. We chose to do the withdrawal as instantaneous for computational simplicity. Making the withdrawal gradual should not affect our results since each neuron has already selected their postsynaptic targets. Figure S3 of Text S1 demonstrates how axon remodeling works in the simulation.

### Neuronal Death

For some simulations, neurons were either allowed to die through a Poisson process or they were “lesioned” in mass. Neuronal death was simulated by selecting a neuron and randomizing its output synaptic connections, giving it a probability of 0.1 of having active connections and 0.9 of silent connections. Input connections were similarly randomized with an additional rule: if the input was a supersynapse, then the presynaptic neuron would be made unsaturated. Dead neurons are immediately available again in the free pool, but because the memory of its synaptic strengths was erased, it acts as an entirely different neuron.

## Supporting Information

Text S1 Materials and methods.
(0.04 MB DOC)

Figure S1 Modification curve for STDP. The amount and direction by which a synapse will change its strength depends upon the spike times of the pre- and postsynaptic neurons. The modification curve shows the amount per spike pair. The ovals show values from simulation. When the presynaptic neuron spikes before the postsynaptic neuron (defined as negative Δt), the synaptic strength increases in strength (LTP). When the presynaptic neuron spikes after the postsynaptic neuron, the synaptic strength decreases in strength (LTD). The percent change in LTP is based on a constant value *G* ^LTP^, whereas the change in LTD is based on the current synaptic strength. The modification curve is linear for close spikes, when the absolute value of Δt is less than about 5 ms, and is an exponential decay beyond that, see the Materials and Methods section of the main text for the values used.
(0.28 MB TIF)

Figure S2 Demonstration of silencing and activating synapses. (A) A diagram of the network used in the simulation. There are five neurons (gray ovals) in total. Four of the neurons are in the training set and receive external synaptic excitation (black tees); the external excitation induces them to spike synchronously. Three of the four training neurons (bottom three) make active synaptic connections (solid straight black arrows) onto the fifth neuron (right oval). One of the four (top left) makes a silent synaptic connection (dashed straight arrow labeled *G* ^1^) onto the fifth neuron; when this neuron spikes, it will not excite the fifth neuron. The fifth neuron makes a reciprocal active synaptic connection (solid curved black arrow labeled *G* ^2^). (B) The plot shows the trajectory of the synaptic strengths *G* ^1^ and *G* ^2^ as a function of trials. In each trial, the left neurons were given external excitation, inducing them to spike. When the lower left three neurons spike, they bias the right neuron to spike after all the left neurons; therefore, those synapses undergo LTP, including the silent synapse *G* ^1^; hence, its strength (unfilled ovals) increases. Near trial 80, *G* ^1^ potentiates above the threshold value (gray dashed line) and the synapse becomes active (black filled ovals). The opposite occurs for *G* ^2^; since the left neurons spike before the right one, *G* ^2^ undergoes LTD; hence, its strength (filled rectangles) decreases. Near Trial 50, *G* ^2^ depresses below the threshold value, and the synapse becomes silent (unfilled rectangles). (C) The final configuration of the network after 150 trials. *G* ^1^ is active, while *G* ^2^ is silent.
(0.29 MB TIF)

Figure S3 Axon remodeling in a simulation of 5 neurons. The trajectory of four synaptic strengths emanating from a single presynaptic neuron (upper). The neuron is induced to spike early in the trial; therefore, the primary induction between it and the other four neurons is LTP. The arrows and letters correspond to snapshots of the synaptic network (lower). (A) The network starts with four active synapses with random initial strengths. (B) One of the synapses, *G* ^3^ (thick black arrow), goes above Θ^S^, making it a supersynapse. At this point, no remodeling occurs. (C) A second synapse, *G* ^1^ , goes above Θ^S^. Now the neuron is saturated (the number of supersynapses per neuron for this demonstration was set to 2 for illustrative purposes). (D) A saturated neuron withdraws the other axon branches, leaving only the supersynapses.
(0.29 MB TIF)

Movie S1 The movie shows the growth of the network from Figure 3 of the main text. The topology of the supersynaptic network was captured every 2500 trials for 250000 trials and played at a frame rate of 2 frames per second; group assignment was set as in Figure 3 of the main text. At the start of the movie, only the training neurons are shown (column of gray ovals). The network then begins to grow by recruiting neurons into successive groups at the end of the chain. Note that the topology of the groups preceding the end of the chain is stable during growth, making occasional adjustments. Around 35 seconds into the movie (trial 180000), the growth of the chain ceases, and a cycle (long blue arrows) is formed. After that point, the topology remains steady with just a few rearrangements.
(0.93 MB MOV)
