# Supplementary material for: Development of Neural Circuitry for Precise Temporal Sequences through Spontaneous Activity, Axon Remodeling, and Synaptic Plasticity
Source: PLoS One. 2007 Aug 8;2(8):e723. doi: 10.1371/journal.pone.0000723 (PMC1933597; doi:10.1371/journal.pone.0000723)
Supplement: Text S1 — Materials and methods. (0.04 MB DOC) [file pone.0000723.s001.doc]

# Materials & Methods S1

# Development of neural circuitry for precise temporal sequences through spontaneous activity, axon remodeling, and synaptic plasticity

**Joseph K. Jun and Dezhe Z. Jin**

In the Materials and Methods section of the main text, we described in detail the implementation of the simulations. Here, we show figures that illustrate the basic concepts (Figures S1, S2, and S3). Finally, we describe in detail the algorithm used to place neurons into groups in Figures 3, 7, 9A, 10A, and Movie S1.

*Spike Timing Dependent Plasticity (STDP).* Figure S1 shows the STDP kernel used to evaluate the amount of potentiation or depression per spike pair as a function of spike timing difference between the pre- and postsynaptic neuron. In our simulations, long term potentiation (LTP) is additive whereas long term depression (LTD) is multiplicative. Therefore, in the positive (LTD) region, the value of the curve is percent change of current synaptic value. On the other hand, in the negative (LTP) region, the value of the curve is percent change of a constant value, $G_{\text{LTP}}$, which is a parameter in the model.

*Synapse Silencing and Activation.* Figure S2 shows a small cartoon simulation that depicts the activation of one synapse and the silencing of another. Four synchronous neurons synapse onto a fifth neuron (Figure S2A); one of the four, however, has a silent synapse onto the fifth neuron, labeled $G_{1}$; the fifth neuron makes a reciprocal active synapse back onto the first, labeled $G_{2}$. Because the synchronous group is driven to spike regularly, three of the synchronous neurons will bias the fifth neuron to spike after them. Therefore synapse $G_{1}$ strengthens due to LTP and eventually cross threshold and activate (Figure S2B). The opposite occurs to synapse $G_{2}$, which weakens due to LTD and eventually goes below threshold and silences.

*Axon Remodeling.* Figure S3, shows another cartoon simulation that demonstrates the axon remodeling process. A single presynaptic neuron makes synapses onto four postsynaptic targets. The synapses are labeled $G_{1}$ through $G_{4}$, and each neuron is limited to 2 supersynapses. When one of the synapses, $G_{3}$, goes superthreshold (Figure S3 upper), the axons of that neuron do not remodel. If a second synapse, $G_{1}$, goes superthreshold and saturates the neuron, then the neuron retracts its remaining synapses (Figure S3 lower).

*Drawing Topology.* Here, we describe the procedure used to draw the synaptic topology shown in Figures 3, 7, 9A, and 10A and Movie S1. The key to drawing the topology is to position each neuron in the approximate order they will spike. Neurons that spike synchronously should be placed in the same group, and groups of neurons that spike later should be placed in higher group numbers. This ranking can be approximated by knowing which neurons make supersynaptic connections to each other. Since the training neurons (TN) are the start of the chain, they are set as the first group; any neurons receiving connections from them are in the second group, etc. We accomplish this ranking as follows. All TN were set as the first group. Choose a TN and look at any of its superthreshold postsynaptic targets, i.e. supersynapses (SS), and set its rank to two. Follow any of that neuron’s SS to be rank three, etc. The first pass will encounter only unranked neurons. Subsequent passes will find some SS targets already ranked. When this happens, the target’s rank is changed only if it will be assigned a lower number than it already has. The recursive procedure continues to move up synapses until a dead end is reached, i.e. a neuron that has no SS or has no SS targets that would change rank. Whenever this happens, the procedure moves back one neuron and follows a different SS until another dead end. The algorithm stops when all possible paths have been visited.

The algorithm described above finds the shortest path (in number of synapses) from the TN to any neuron. This arrangement may be a poor representation of spike order since a single SS can pull a neuron into a low rank even if the majority of its afferent synapses originate from a higher rank. In other words, a single SS may be insufficient to induce the postsynaptic neuron to spike; it may require several neurons to spike in close temporal proximity to one another in order for the target to spike. Therefore, assigning neurons the lowest rank they can attain based on any single synapse often produces a poor representation of the spike order in the chain. To correct for this, the initial recursive embedding is corrected by looking at every neuron’s afferent synapses. Incoming SS may originate from neurons with different ranks. The rank that makes the most number of SS onto a synapse decides that neuron’s rank, setting it to one higher than the majority rank value. In the case of a tie, the lower value is chosen. After each neuron is assigned a position in the network, the *Combinatorica* package [1] of the commercial software *Mathematica* is used to draw the topology.

**References**

1 Pemmaraju S, Skiena S (2003) Computational Discrete Mathematics: Combinatorics and Graph Theory with Mathematica. Cambridge: Cambridge University Press.
