# Supplementary figures and images for: Development of Neural Circuitry for Precise Temporal Sequences through Spontaneous Activity, Axon Remodeling, and Synaptic Plasticity
Source: PLoS One. 2007 Aug 8;2(8):e723. doi: 10.1371/journal.pone.0000723 (PMC1933597; doi:10.1371/journal.pone.0000723)

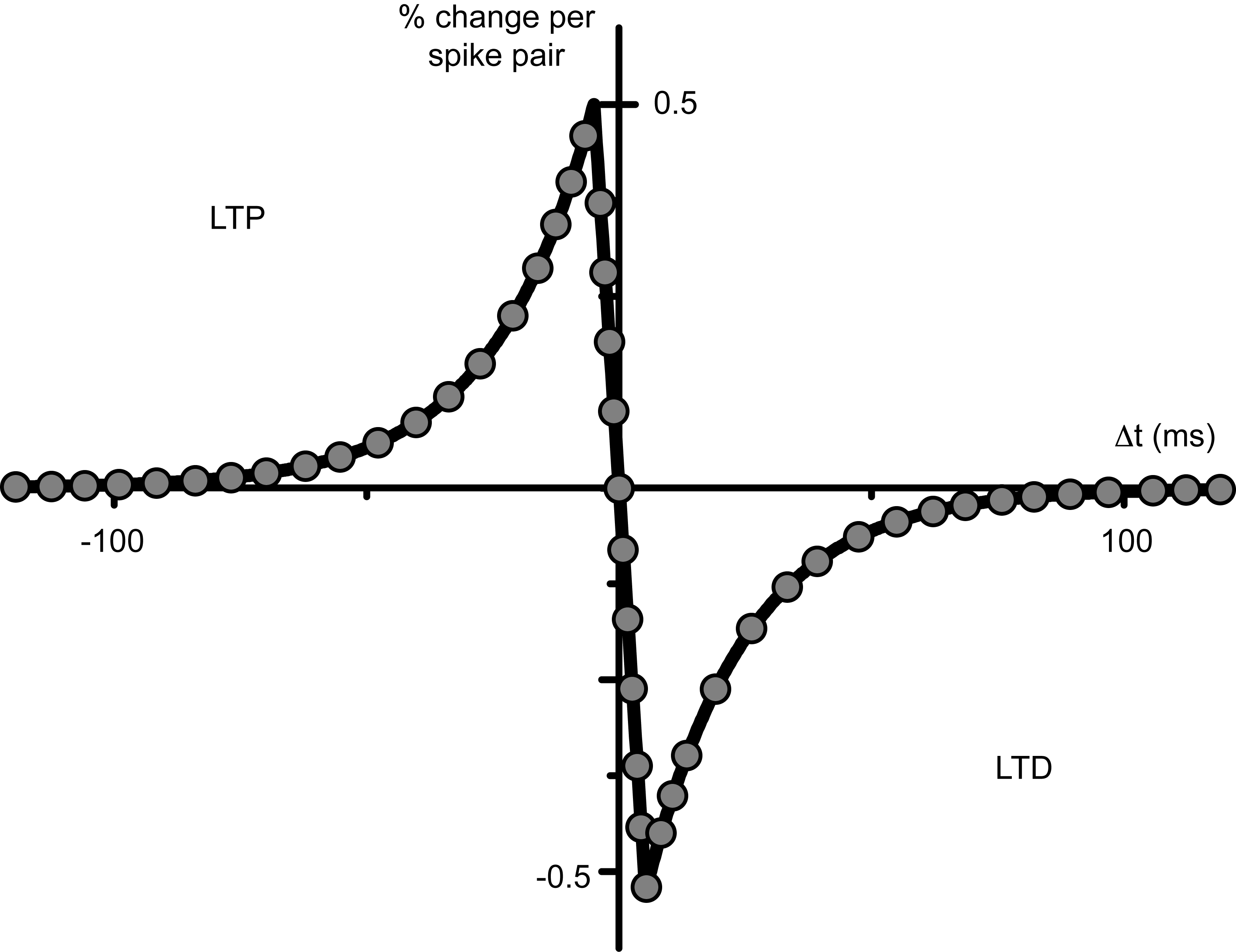

Supplement: Figure S1 — Modification curve for STDP. The amount and direction by which a synapse will change its strength depends upon the spike times of the pre- and postsynaptic neurons. The modification curve shows the amount per spike pair. The ovals show values from simulation. When the presynaptic neuron spikes before the postsynaptic neuron (defined as negative Δt), the synaptic strength increases in strength (LTP). When the presynaptic neuron spikes after the postsynaptic neuron, the synaptic strength decreases in strength (LTD). The percent change in LTP is based on a constant value G LTP, whereas the change in LTD is based on the current synaptic strength. The modification curve is linear for close spikes, when the absolute value of Δt is less than about 5 ms, and is an exponential decay beyond that, see the Materials and Methods section of the main text for the values used. (0.28 MB TIF) [file pone.0000723.s002.tif]

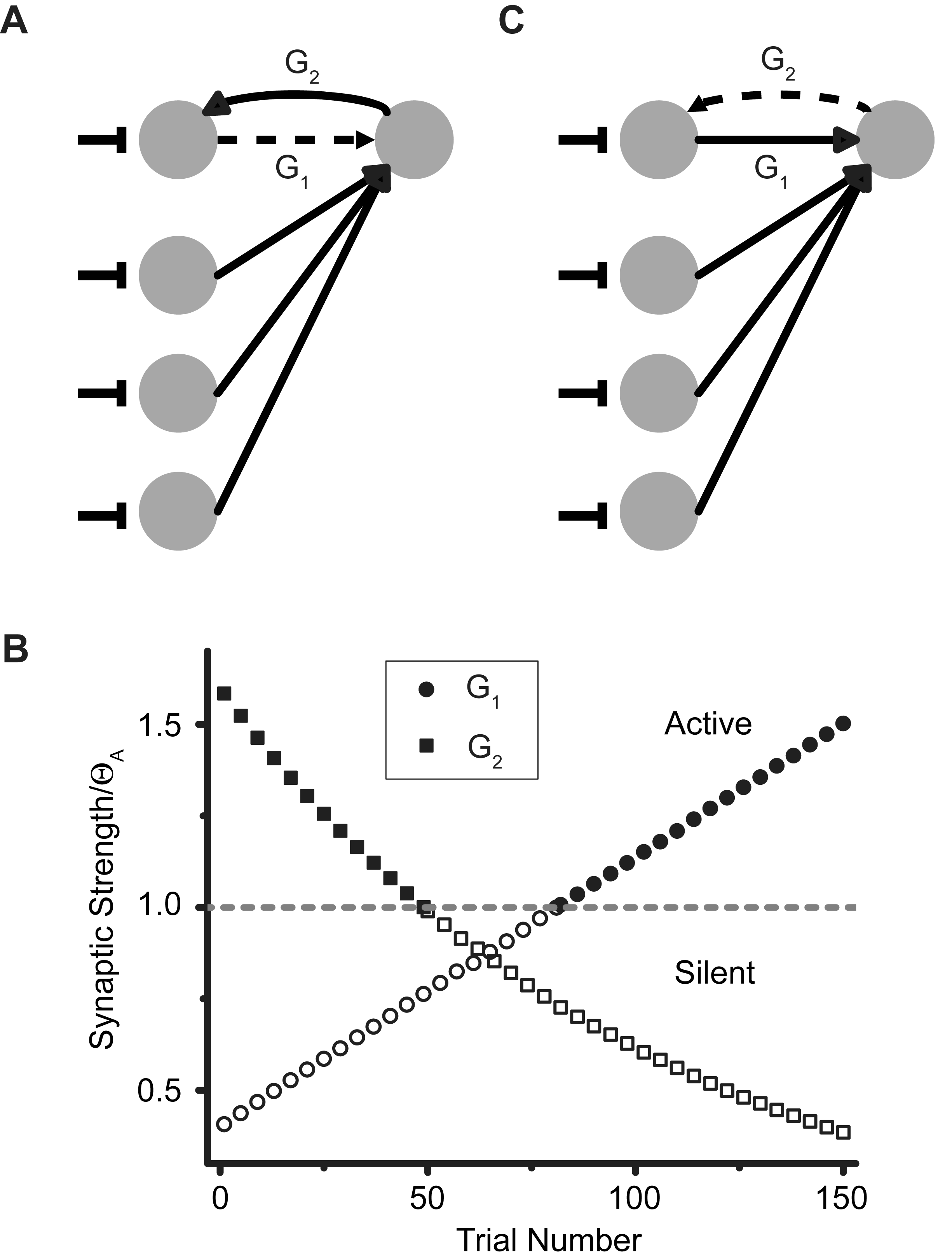

Supplement: Figure S2 — Demonstration of silencing and activating synapses. (A) A diagram of the network used in the simulation. There are five neurons (gray ovals) in total. Four of the neurons are in the training set and receive external synaptic excitation (black tees); the external excitation induces them to spike synchronously. Three of the four training neurons (bottom three) make active synaptic connections (solid straight black arrows) onto the fifth neuron (right oval). One of the four (top left) makes a silent synaptic connection (dashed straight arrow labeled G 1) onto the fifth neuron; when this neuron spikes, it will not excite the fifth neuron. The fifth neuron makes a reciprocal active synaptic connection (solid curved black arrow labeled G 2). (B) The plot shows the trajectory of the synaptic strengths G 1 and G 2 as a function of trials. In each trial, the left neurons were given external excitation, inducing them to spike. When the lower left three neurons spike, they bias the right neuron to spike after all the left neurons; therefore, those synapses undergo LTP, including the silent synapse G 1; hence, its strength (unfilled ovals) increases. Near trial 80, G 1 potentiates above the threshold value (gray dashed line) and the synapse becomes active (black filled ovals). The opposite occurs for G 2; since the left neurons spike before the right one, G 2 undergoes LTD; hence, its strength (filled rectangles) decreases. Near Trial 50, G 2 depresses below the threshold value, and the synapse becomes silent (unfilled rectangles). (C) The final configuration of the network after 150 trials. G 1 is active, while G 2 is silent. (0.29 MB TIF) [file pone.0000723.s003.tif]

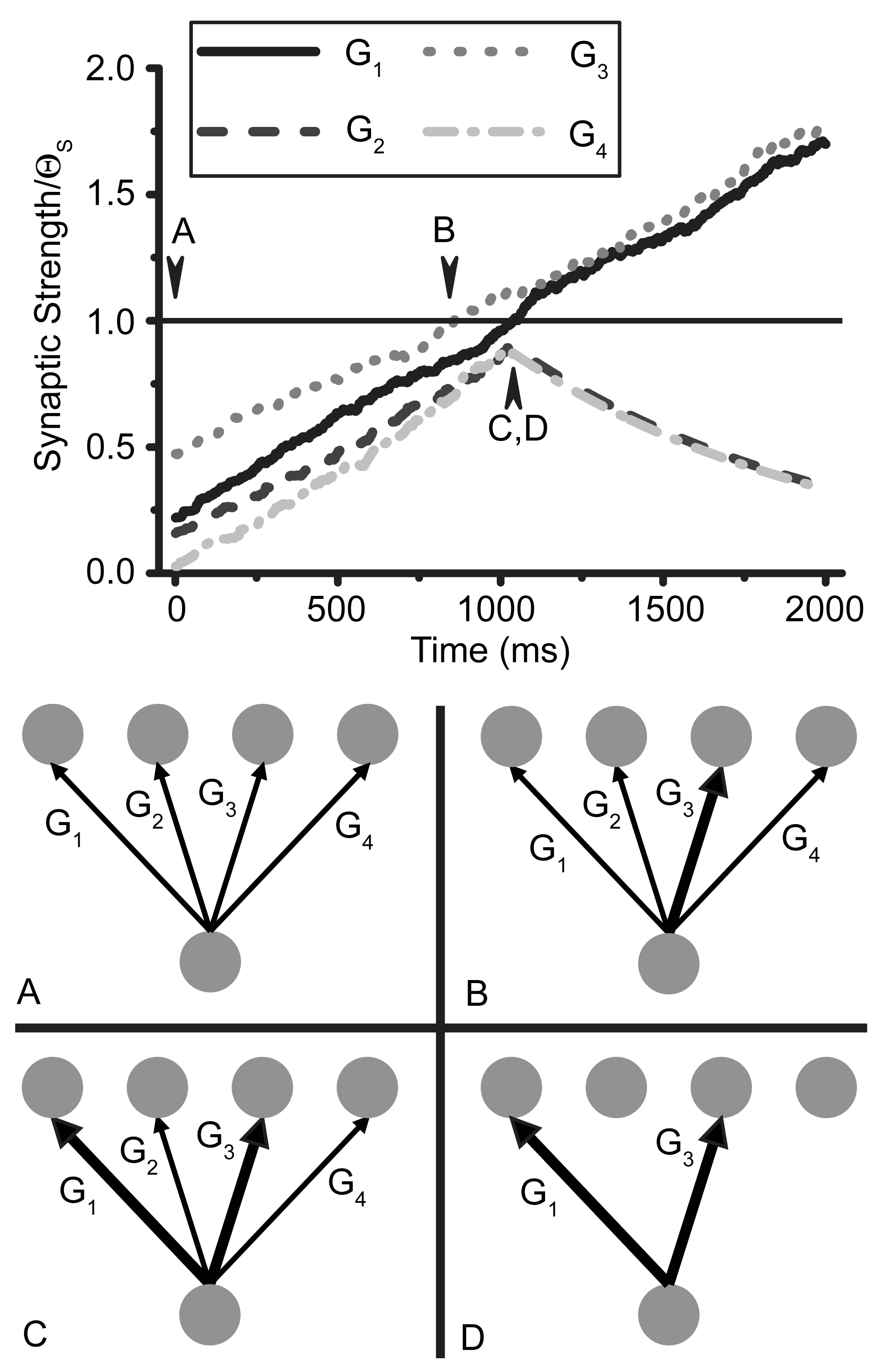

Supplement: Figure S3 — Axon remodeling in a simulation of 5 neurons. The trajectory of four synaptic strengths emanating from a single presynaptic neuron (upper). The neuron is induced to spike early in the trial; therefore, the primary induction between it and the other four neurons is LTP. The arrows and letters correspond to snapshots of the synaptic network (lower). (A) The network starts with four active synapses with random initial strengths. (B) One of the synapses, G 3 (thick black arrow), goes above ΘS, making it a supersynapse. At this point, no remodeling occurs. (C) A second synapse, G 1 , goes above ΘS. Now the neuron is saturated (the number of supersynapses per neuron for this demonstration was set to 2 for illustrative purposes). (D) A saturated neuron withdraws the other axon branches, leaving only the supersynapses. (0.29 MB TIF) [file pone.0000723.s004.tif]
